# Comparative Atlas of SARS-CoV-2 Substitution Mutations: A Focus on Iranian Strains Amidst Global Trends

**DOI:** 10.3390/v16081331

**Published:** 2024-08-20

**Authors:** Mohammad Hadi Abbasian, Karim Rahimian, Mohammadamin Mahmanzar, Saleha Bayat, Donna Lee Kuehu, Mahsa Mollapour Sisakht, Bahman Moradi, Youping Deng

**Affiliations:** 1 Department of Medical Genetics, National Institute for Genetic Engineering and Biotechnology, Tehran 1497716316, Iran; mh_abbasian@nigeb.ac.ir; 2 Institute of Biochemistry and Biophysics (IBB), University of Tehran, Tehran 14174, Iran; karimrahimian@gmail.com; 3 Department of Bioinformatics, Kish International Campus University of Tehran, Kish 7941639982, Iran; mahmanza@hawaii.edu; 4 Department of Quantitative Health Sciences, John A. Burns School of Medicine, University of Hawaii at Manoa, Honolulu, HI 96813, USA; dkuehu@hawaii.edu; 5 Department of Biology & Research Center for Animal Development Applied Biology, Mashhad Branch, Islamic Azad University, Mashhad 9187147578, Iran; saba80012@gmail.com; 6 Faculty of Pharmacy, Biotechnology Research Center, Tehran University of Medical Sciences, Tehran 1936893813, Iran; mmollapour@farabi.tums.ac.ir; 7 Department of Biology, Faculty of Sciences, Shahid Bahonar University of Kerman, Kerman 7616913439, Iran; bahmanmoradi.biocan1400@gmail.com

**Keywords:** coronavirus, SARS-CoV-2, COVID-19, vaccines, genomics, genetic variability, mutation hotspot

## Abstract

Background: Severe acute respiratory syndrome coronavirus 2 (SARS-CoV-2) is a new emerging coronavirus that caused coronavirus disease 2019 (COVID-19). Whole-genome tracking of SARS-CoV-2 enhanced our understanding of the mechanism of the disease, control, and prevention of COVID-19. Methods: we analyzed 3368 SARS-CoV-2 protein sequences from Iran and compared them with 15.6 million global sequences in the GISAID database, using the Wuhan-Hu-1 strain as a reference. Results: Our investigation revealed that NSP12-P323L, ORF9c-G50N, NSP14-I42V, membrane-A63T, Q19E, and NSP3-G489S were found to be the most frequent mutations among Iranian SARS-CoV-2 sequences. Furthermore, it was observed that more than 94% of the SARS-CoV-2 genome, including NSP7, NSP8, NSP9, NSP10, NSP11, and ORF8, had no mutations when compared to the Wuhan-Hu-1 strain. Finally, our data indicated that the ORF3a-T24I, NSP3-G489S, NSP5-P132H, NSP14-I42V, envelope-T9I, nucleocapsid-D3L, membrane-Q19E, and membrane-A63T mutations might be responsible factors for the surge in the SARS-CoV-2 Omicron variant wave in Iran. Conclusions: real-time genomic surveillance is crucial for detecting new SARS-CoV-2 variants, updating diagnostic tools, designing vaccines, and understanding adaptation to new environments.

## 1. Introduction

Severe acute respiratory syndrome coronavirus 2 (SARS-CoV-2) is an RNA virus first identified in Wuhan, China [1]. SARS-CoV-2 disseminated rapidly around the world, and the World Health Organization (WHO) officially declared coronavirus disease 2019 (COVID-19) a global pandemic in March 2020 [2]. As of June 2024, SARS-CoV-2 has been found to be the cause of approximately 775 million confirmed cases and over 7 million deaths globally [3] (https://covid19.who.int/, accessed on 10 August 2024). The first COVID-19 case in Iran was confirmed in Qom on 19 February 2020 [4]. As of 4 October 2023, there have been 7,617,752 confirmed cases and 146,410 deaths reported in Iran.

Throughout the COVID-19 pandemic, genomics has played a crucial role in tracking the SARS-CoV-2 virus and gaining a better understanding of genetic susceptibility to the disease [5]. Previous studies have shown that epidemiological data combined with SARS-CoV-2 whole-genome sequence analysis are pivotal for public health decision making [6,7]. The first SARS-CoV-2 sequence was made publicly available in January 2020 [8]. The Global Initiative on Sharing All Influenza Data (GSAID) (https://www.gisaid.org) is a global science initiative that facilitates analysis sequences of SARS-CoV-2 and monitors emerging new variants [9,10,11]. Sharing SARS-CoV-2 genomic data through different databases such as the SARS2Mutant database (http://sars2mutant.com/), the European COVID-19 Data Platform (https://www.covid19dataportal.org/), and Nextstrain (https://nextstrain.org/) led to the discovery of mutations and conserved regions, thereby accelerating coronavirus research [12,13,14].

**Figure 1:**
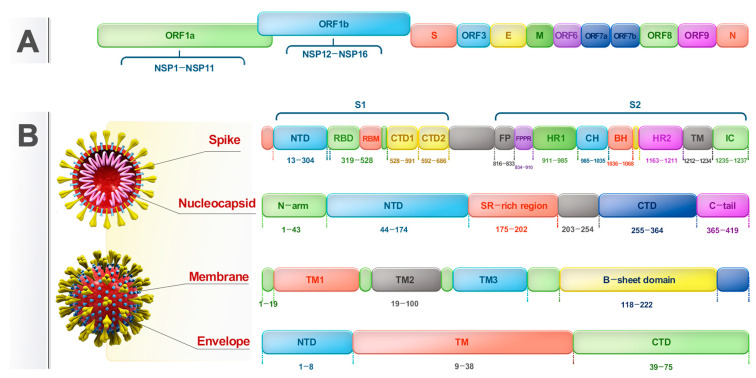
Schematic representation of SARS-CoV-2 genome. (**A**) 29,903 nucleotide base pairs of SARS-CoV-2 single-stranded RNA genome. (**B**) SARS-CoV-2 four structural proteins.

The SARS-CoV-2 genome, which is one of the largest among RNA viruses, ranges from 29.8 to 29.9 kb and is organized into a small nucleocapsid (N) protein (Figure 1). The SARS-CoV-2 genome is arranged from 5′ to 3′ untranslated regions (UTRs) as non-structural genes (ORF1a/ORF1b replicase gene) and structural genes such as spike (S), envelope (E), membrane (M), and N and accessory genes (ORF3a, ORF6, ORF7a, ORF7b, ORF8, ORF9b, and ORF9c) [12]. The S glycoprotein recognizes the ACE-2 receptors of the host cells [13]. M protein helps to enclose mature virus particles in a membrane, and the virion particles are assembled by the E protein. The SARS-CoV-2 genome is composed of 16 non-structural proteins (NSPs). The NSPs comprise various viral cysteine proteases such as main proteinase (NSP5), putative transmembrane domain (NSP6), RNA-dependent RNA polymerase (NSP12), helicase (NSP13), and 2′-O-methyltransferase (NSP16) that play critical roles in viral RNA transcription and replication [14,15].

It was evident that the pandemic could only be controlled with efficacious vaccines [16]. Vaccine development is the first and most favorable response to control the devastating impact of the COVID-19 pandemic. Thus, several new vaccine platforms such as mRNA, vector-based, inactivated, and protein-based vaccines were developed [17,18,19]. However, the emergence of variants of concern (VOCs) poses challenges to vaccine efficacy, because of mutations such as D614G, L452R, P681R, and E484K in the S protein which potentially reduce vaccine effectiveness [20]. Vaccines are designed to induce immune responses against SARS-CoV-2 S glycoprotein [21]. Accumulating evidence shows that VOCs contain different mutations in the SARS-CoV-2 genome. For instance, VOCs containing the D614G, L452R, P681R, and E484K mutations in the S protein potentially affect the transmissibility and the virulence of SARS-CoV-2, which can reduce vaccine efficacy [22,23,24]. The Delta and Omicron variants, in particular, have been shown to diminish protection against COVID-19 [25,26]

Furthermore, circulating SARS-CoV-2 variants of interest (VOIs) and variants under monitoring (VUMs) are essential for close monitoring. For instance, the Delta variant was initially considered a VOI, but its rapid global spread led the WHO to reclassify it as a VOC in May 2021. These variants may be associated with increased transmissibility in the future and potential implications for vaccine efficacy [27]. Therefore, continuous monitoring of SARS-CoV-2 mutations is critical for better control of the COVID-19 pandemic.

In Iran, several studies have investigated SARS-CoV-2 genome sequences to understand its origin, transmission dynamics, and impact on evolution and disease spread. Eslami et al. identified a unique mutation, E1202Q, in the HR2 subdomain, which facilitates virus membrane fusion [28]. Another study demonstrated that the S mutation D614G increased infectivity and transmission of SARS-CoV-2 [29]. Additionally, common mutations were found in the ORF1ab, S, N, intergenic, and ORF7 regions [30]. These findings contribute to our understanding of the genetic changes in the SARS-CoV-2 genome in Iran.

The present study provides the most comprehensive description of the SARS-CoV-2 epidemic in Iran based on a genomic epidemiology approach. In total, 3368 Iranian SARS-CoV-2 sequences were compared to approximately 15,669,529 worldwide genomes from January 2019 to June 2023. NSP12-P323L, ORF9c-G50N, NSP14-I42V, membrane-A63T, Q19E, and NSP3-G489S were the most common mutations. Over 94% of the SARS-CoV-2 genome in Iran (including NSP7, NSP8, NSP9, NSP10, NSP11, and ORF8) remained unchanged compared to the Wuhan-Hu-1 strain. Mutations such as ORF3a-T24I, NSP3-G489S, NSP5-P132H, NSP14-I42V, envelope-T9I, nucleocapsid-D3L, membrane-Q19E, and membrane-A63T may contribute to the recent surge in the SARS-CoV-2 Omicron variant in Iran.

## 2. Materials and Methods

### 2.1. Sequence Source

**Figure 2:**
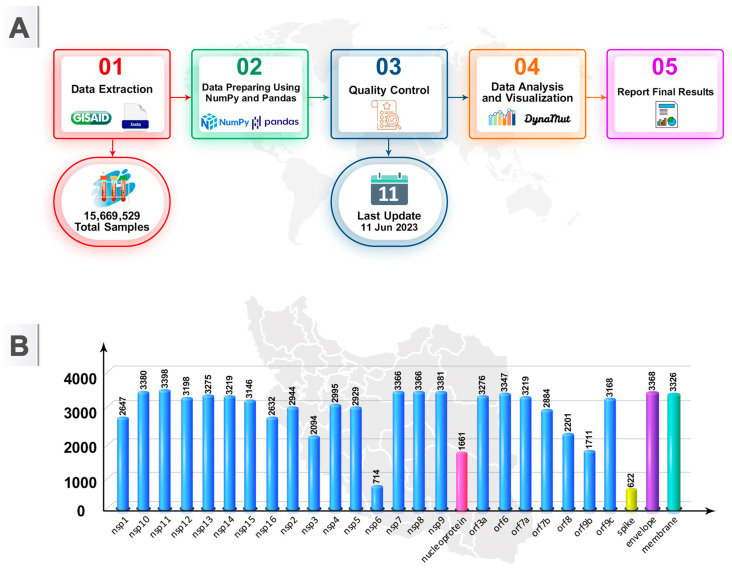
Study design. (**A**) Flowchart of methods involved in this study. (**B**) Number of SARS-CoV-2 genome samples for each gene.

This study evaluated the complete dataset of SARS-CoV-2 amino acid (AA) sequences (AASs). All AASs were compared to the Wuhan-2019 reference sequence ‘EPI_ISL_402124’. AASs of SARS-CoV-2 from various geographic locations in Iran were retrieved from the GISAID database [9,11]. We have access to this database with the permission of John A. Burns School of Medicine. The study design and flowchart of methods are summarized in Figure 2. Figure 2A illustrates the flowchart of methods used in this study, while Figure 2B shows the distribution of SARS-CoV-2 genome samples across different genes.

### 2.2. Sequence Analyses and Exclusion Criteria

Python 3.8.0 software was utilized to preprocess FASTA files. Mutations were identified when any difference was found between the SARS-CoV-2 sequences and the reference, within the location and the substituted AA reported. Non-human samples and those with more or less than the length of SARS-CoV-2 genes and samples containing non-specified AAs (reported as X) were omitted. The whole process was optimized by applying ‘Numpy’ and ‘Pandas’ libraries, as previously described [31]. Briefly, for detection of mutations in reference and sample sequences, we used ‘Refseq’ and ‘seq’, respectively. For refitem, seqitem in zip (refseq, seq) If (refitem! = seqitem) report a new mutant. After extracting genome sequences of SARS-CoV-2, each sample’s continent name and geographical coordinates were obtained and reported using pycountry-convert 0.5.8 software and ‘Titlecase’ library in Python to draw global prevalence maps of mutations. We employed the proportions Z-test to evaluate the statistical significance of the differences in mutation rates between the Iranian and worldwide samples. Each mutation’s rate was tested independently. A *p*-value threshold of 0.05 was used to determine statistical significance. The effects of stability and flexibility of protein changes were analyzed using the DynaMut server [32]. This server defined the point mutations as stabilizing (ΔΔG value was described as ≥0) and destabilizing (ΔΔG was illustrated as <0). PDB structures were downloaded from SARS-CoV-2 3D database [33]. Figures were drawn using VMD version 1.9.3 and GraphPad Prism, version 8.0.2.

## 3. Results

### 3.1. Recurrent Mutations and Hotspots and Conserved Domains in the SARS-CoV-2 Genome

We compared 3368 SARS-CoV-2 protein sequences from Iranian samples to approximately 15,669,529 global SARS-CoV-2 genomes from the period between January 2019 and June 2023. The sequences from the GISAID database were compared with the Wuhan-Hu-1 reference strain (Accession NC_045512). Among the Iranian samples, 18 mutations were present in more than 40% of the sequences (Table 1). Additional amino acid substitutions were found in these 18 mutations in Iran as well as globally and are listed in Supplementary Tables S6 and S7. Our analysis revealed significant differences in mutation rates between Iranian and worldwide samples across all genes and mutations studied. The mutations displayed varying degrees of prevalence disparity, with some showing dramatic differences between the two populations. Notable mutations with highly significant *p*-values included P323L (in NSP12), G50N (in ORF9c), and T492I (in NSP4), among others. 

**Figure 3:**
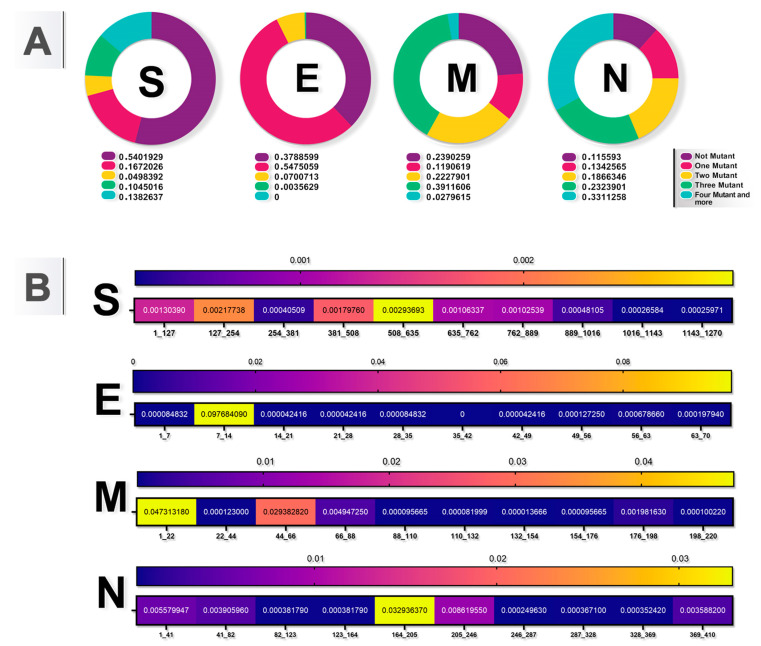
Conserved and hotspot regions in structural proteins of SARS-CoV-2. (**A**) Pie charts show the proportion of the mutations in S, E, M, and N proteins of SARS-CoV-2. (**B**) Heat map of genome conservation data showing the regions that were differentially mutated in S, E, M, and N proteins of SARS-CoV-2.

Figure 3A illustrates the distribution of mutant variants in different structural proteins of SARS-CoV-2. Each chart represents the proportion of non-mutant and various mutant categories (one mutation, two mutations, three mutations, and four or more mutations) within the S, E, M, and N proteins. Figure 3B depicts a heat map of genome conservation data, highlighting regions that exhibit differential mutations in the S, E, M, and N proteins of SARS-CoV-2.

Our analysis identified that just 54.0% of the SARS-CoV-2 S protein did not have any mutations (Figure 3A). More importantly, 13.8% of the SARS-CoV-2 genome had four or more mutations. Our results revealed that two residues (508–635 and 127–254) are hotspot regions in the S protein (Figure 3B). Common mutations were observed including D614G (50.9%), S477N (32.2%), T478K (25.4%), H655Y (24.7%), N501Y (24.4%), P681H (24.1%), and E484 (24.1%) (Table 1 and Supplementary Table S4). 

In our analysis, 37.8% of the SARS-CoV-2 E protein had no mutations (Figure 3A). 

The most common mutations found in the E protein were T9I (61.6%), T11A (6.71%), and D72G (0.32%) (Table 1 and Supplementary Table S1). M protein is a 221-amino-acid-long viral assembly protein with three transmembrane helices and a cytoplasmic C-terminal domain. Based on our results, our analysis shows that 23.9% of the M protein had no mutations. The most common mutations in our sample were A63T (64.5%), Q19E (63.7%), D3N (23.6%), and D3G (16.0%), with I82T (7.03%) and I73M (3.63%) (Table 1 and Supplementary Table S2). 

The N protein of SARS-CoV-2 has 419 amino acids, involves different cellular processes, and plays a key role in the viral life cycle and host infection [34]. The N protein is involved in the packing of RNA, the release of virus particles, and the formation of the ribonucleoprotein core [35]. In our study, 11.5% of the N protein had no mutations and 33.1% had four or more mutations (Figure 3A). The most frequent mutations were found in A63T (64.5%), Q19E (63.7%), D3N (23.6%), and D3G (16.0%), with I82T (7.03%) and I73M (3.63%) also being notable (Table 1 and Supplementary Table S2). Among the N mutations, R203K (52.1%), G204R (51.9%), S413R (22.6%), D377Y (15.7%), D63G (15.5%), G215C (13.7%), S235F (13.6%), S235F (13.5%), D3L (13.0%), and S194L (9.63%) were the most common in our samples (Table 1 and Supplementary Table S3). 

**Figure 4:**
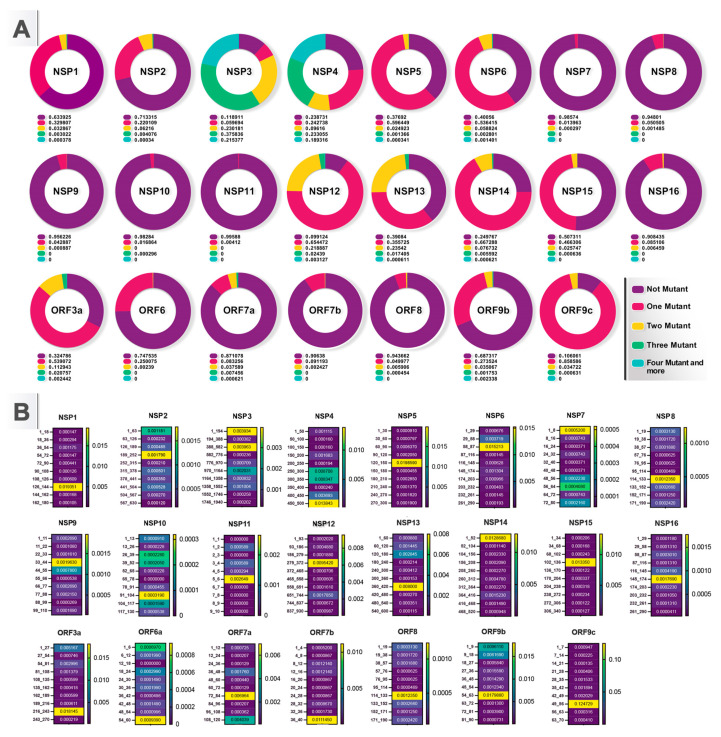
Conserved and hotspot regions in non-structural proteins of SARS-CoV-2. (**A**) Pie charts show the proportion of the mutants in SARS-CoV-2. (**B**) Heat map of genome conservation data showing the regions that were differentially mutated in SARS-CoV-2.

In our study, 9.91% of NSP12 showed no mutations, with hotspots at 279_372 and conserved regions at 186–279 and 837–930 (Figure 4). The most frequent mutations found in NSP12 were P323L (88.2%), G671S (15.6%), and G137C (2.72%). Conserved regions and common mutations in other SARS-CoV-2 genes are detailed in Supplementary File S1, providing a comprehensive analysis of conserved regions and common mutations across various SARS-CoV-2 genes [15,36,37,38,39,40,41,42,43,44,45,46,47,48,49,50,51,52,53,54,55,56,57,58,59,60,61,62,63,64,65,66,67,68,69,70,71,72,73,74,75,76,77,78,79,80,81,82,83,84,85,86,87,88,89,90,91,92,93,94,95,96,97,98,99,100,101,102,103,104].

### 3.2. Chronological Trend of Common SARS-CoV-2 Mutations

Detecting and identifying circulating new SARS-CoV-2 variants, along with assessing their consequences, are crucial for managing and controlling the spread of VOCs. Additionally, they are essential for tracking and predicting VOIs and VUMs.

**Figure 5:**
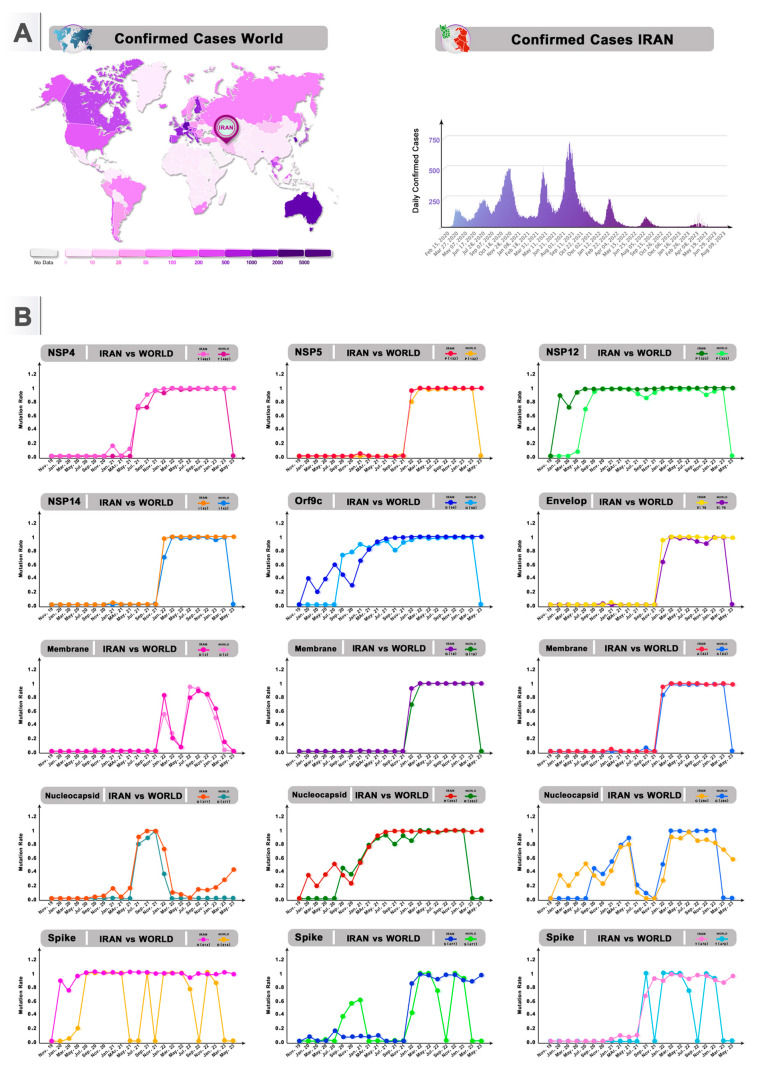
Timeline of common mutations in circulating SARS-CoV-2. (**A**) Confirmed COVID-19 cases around the world and in Iran. (**B**) Trends in common mutations in Iran and around the world from January 2020 to June 2023.

To elucidate how the amino acid changes in the SARS-CoV-2 genome are responsible for different outbreak waves, we explore the frequency of the top mutations in Iran from January 2020 to June 2023 (Figure 5B, and Supplementary Table S5).

The D614G mutation was first identified in January 2020, and by the end of March 2020 it had increased in frequency worldwide and became dominant worldwide until now. In late March 2020, the first S protein mutations in D614 were detected in Iran, and they were stable mutations until February 2023 (Figure 5B). Our study reveals that S477 was initially detected in Iran in October 2020 and exhibited an increasing trend from October 2020 to February 2021. The first amino acid substitution in T478 was detected on 31 January 2020 worldwide; then, in August 2021, the T478 mutation dramatically increased in the following months. T478K was first detected in Iran in August 2021. N501Y/R was first detected in Iran in March 2021 followed by a marked increase in December 2021. The P681R/H mutation was initially detected in April 2021 and then increased from October 2021 to March 2022. Mutations D3, R203, and G204 were observed in Iran between May and August 2020 and increased in prevalence by September and December 2020, remaining stable for several months (Figure 5B).

The NSP12 P323 mutation emerged in May 2020 and gradually stabilized in the following months (Figure 5B). The Omicron variant, characterized with over 32 mutations compared to the original virus, was first identified in South Africa in November 2021 and in Iran in December 2021. The Omicron variant is characterized by more than 32 mutations compared to the original virus: NSP3 K38R, V1069I, Δ1265, L1266I, A1892T, NSP4 T492I, NSP5 P132H, NSP6 Δ105–107, A189V, NSP12 P323L, and NSP14 I42V, E T9I, M D3G, Q19E, and A63T, N P13L, Δ31–33. R203K. G204R [30]. Notably. our results showed several common mutations, such as NSP3 T24I and G489S, NSP5 P132H, NSP14 I42V, E T9I, M D3L Q19E, and A63T, emerged in late November and December in Iran and worldwide and then increased dramatically until March 2022 (Figure 5B). These mutations can be considered a major driver of the COVID-19 surge in the sixth COVID-19 wave in Iran in January 2022.

### 3.3. Stability and Flexibility of Protein Changes

**Figure 6:**
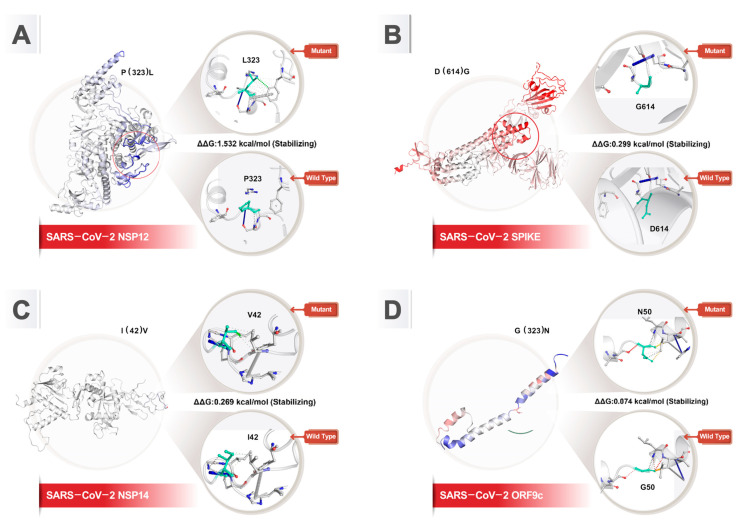
DynaMut prediction of the effects of common SARS-CoV-2 mutations on protein flexibility and stability. Light green represents wild-type and mutant residues of proteins. The flexibility and rigidity of proteins are highlighted in red and blue color, respectively. (**A**) NSP12 mutation P323L; (**B**) spike mutation D614G; (**C**) NSP14 mutation I42V; (**D**) ORF9c mutation G50N.

In this study, we chose P323L, G50N, I42V, and D614G mutations for a more detailed analysis. We studied protein dynamics and stability to elucidate the impact of mutations by performing DynaMut analysis, which combines the calculation of protein stability and dynamics of Bio3D, ENCoM, and DUET methods. Furthermore, the DynaMut server predicts several structure-based methods including SDM [105], mCSM-Stability [106], and DUET [107]. The research outcome for the free energy differences, ΔΔ*G*, showed that P323L, G50N, I42V, and D614G were stable changes with 1.532 kcal/mol, 0.074 kcal/mol, 0.269 kcal/mol, and 0.299 kcal/mol, respectively. Furthermore, P323L and I42V decrease the flexibility of NSP12 and NSP14, respectively (Table 2, Figure 6).

## 4. Discussion

This study analyzed SARS-CoV-2 genomes from Iranian samples collected between January 2020 and June 2022. Previous genomics analysis in Iran reported six mutations that have a frequency of more than 50% in sequences, including D614G, P323L, R203K, G204R, F105F, and C241T [29]. In the study by Sadeghi et al., E T9I, M A63T, Q19E, N R203K, and G204R mutations were detected in SARS-CoV-2 variants compared to mutations during the past waves in Iran [108]. In the United States, top mutations were reported as F106F, S76S, L7L, T85I, P323L, D614G, Q57H, L84S, Y541C, P504L, S24L, R203K, and G204R [94]. Previous genomics analysis in Iran reported that six mutations (D614G, P323L, R203K, G204R, F105F, and C241T) showed a frequency of more than 50% [109]. In the United States, top mutations were reported as F106F, S76S, L7L, T85I, P323L, D614G, Q57H, L84S, Y541C, P504L, S24L, R203K, and G204R [94].

SARS-CoV-2 genomes from Iranian samples exhibit greater diversity and higher variant frequency compared to other Middle Eastern regions. Studies suggest that Iran may have played a significant role in introducing COVID-19 to the rest of the Middle East [110]. In their investigation, Sallam et al. focused on mutations in the S gene of SARS-CoV-2 sequences from the Middle East and North Africa (MENA). They observed that the most common mutation in the entire S region was D614G (435 occurrences), followed by Q677H (8 occurrences) and V6F (5 occurrences) [111]. Obeid et al. conducted a study on 774 SARS-CoV-2 genomic sequences from various regions in Saudi Arabia. They found that the most prevalent variants were the NSP12_P323L mutation (94.9%), followed by the D614G mutation (76%) and the NS3_Q57H mutation (71.4%) [112]. Additionally, during the Delta and Omicron waves at a Saudi tertiary referral hospital, the variants with the highest frequencies were D614G (82.6%), T478K (61.6%), K417N (55.6%), H69del (55.1%), and N440K (50.9%) [113]. In their analysis of SARS-CoV-2 genomic sequences from Eastern Mediterranean Region (EMR) countries, Omais et al. identified ten common non-synonymous mutations. Among these, two substitutions—S_D614G and NSP12_P323L—were predominant across most countries in the region [114].

Within the SARS-CoV-2 S protein, the mutation with glycine at residue 614 (D614) is a highly variable site, and previous studies have indicated that D614G increases the infectivity of the COVID-19 virus. This mutation changes the polar, negatively charged aspartate (D) to the non-polar glycine (G) at residue 614. D614G and D614N were detected in 49% and 0.6% of our samples, respectively. D614G has the second-highest frequency in the United States and has been associated with increased viral replication in primary human upper airway tissues [115]. A global analysis by Abavisani et al. indicated that D614G was the most frequent mutation in the S protein [116]. Within the RBD, S477N is the frequent mutation that has a pivotal role in the binding of the SARS-CoV-2 S protein with the hACE2 receptor [117]. In our samples, the D138Y, N501Y, and E484K mutations were observed at frequencies of 14%, 7%, and 3%, respectively. These mutations were reported in the lineage P.1 and the B.1.1.28 variant [118]. N501Y and E484K occur at the receptor-binding motif (RBM) and increase binding affinity to hACE2 [119]. The N501Y and E484K mutations increased the infectivity while reducing its sensitivity to neutralization by the sera of vaccinated individuals [120]. N501Y has one of the most frequent mutations in RDB and can influence the efficiency of vaccines and drug targeting. The E484K mutation occurs in different variants such as the Delta sublineages B.1.617.2 and B.1.351, and it has been suggested that it reduces antibody neutralization [121]. 

The E protein is a tiny 76–109 amino acid protein. It works as an ion-channeling viroporin, facilitating viral release by damaging host membranes. T9I (61.6%) was a common mutation in Iran. T9I was one of the top mutations worldwide, particularly in the Middle East [122]. T9I was one of the top mutations worldwide. In this mutation, hydrophilic amino acids become hydrophobic, thereby positively modifying the membrane attachment and ER targeting abilities of E protein [86] and destabilizing E protein structure [123]. Rahman et al. analyzed 81,818 sequences of SARS-CoV-2 belonging to 159 countries or territories until 20 August 2020. They found that 1.2% (982/81,818) of strains possessed amino acid substitutions in 63 sites of the E protein. Previous studies showed that 98.8% of the E proteins of globally circulating SARS-CoV-2 strains were conserved [123,124].

The SARS-CoV-2 M gene is highly conserved compared to SARS-CoV-2 (identity: 90.5%; similarity: 98.2%) and Bat and Pangolin isolates [125]. The most common mutation in our sample was A63T (64.5%), and this mutation could potentially affect the stability of the M protein dimer. The Q19E mutation is located in the N-terminal domain and was observed for all the major Omicron subvariants [126]. In our study, the frequencies of D3N and D3G were 23.6% and 16.0%, respectively. The BA.1 and BA.5 subvariants had the N-terminal mutations D3G (aspartic acid to glycine) and D3N (aspartic acid to asparagine), respectively, which may affect the N-myristoylation site at the 3–8 position [57]. 

The N gene is one of the most non-conserved genes in SARS-CoV-2 [127]. The N protein, a multivalent RNA-binding protein, plays a role in genome packaging, host translation interference, and RNA chaperoning [128]. According to our study, the most common mutations in the N protein were R203K and G204R. Accumulation of N gene mutations in the linker and the unstructured regions was also detected in Russian samples [127]. The SR rich-linker has a different role in SARS-CoV-2, including oligomerization, phospho-regulation, and RNA and protein binding [129,130]. Among these, S197L, R203K, and G204R were observed worldwide. In the mutation R203K, both arginine (R) and lysine (K) are positively charged, so this mutation may not effect on the N protein structure and function. However, glycine (G) is a non-polar residue, and its replacement with the positively charged arginine (R) may destabilize the N protein structure [131]. NSP12, the most conserved protein in coronaviruses, is crucial for viral replication and transcription. The NSP12 of SARS-CoV-2 shares 96% sequence identity with SARS-CoV-2 and 71% with MERS-CoV-2 [53]. The SARS-CoV-2 NSP12 also contains a nidovirus-unique N-terminal extension (amino acids 1–397) and a polymerase domain (amino acids 398–919) [132]. Common NSP12 mutations in our sample, P323L, G137C, and G137S, were located in the polymerase domain. The P323L mutation was reported for the first time in Spain on 25 January 2020. The P323L mutation was one of the dominant mutations in the United States [94]. In this mutation, the amino acid leucine is substituted for proline, which may not have an effect on the NSP12 function. However, Wang et al. suggested that this mutation might enhance the transmission capacity of SARS-CoV-2 [94]. Our analysis showed that the NSP12 P323L mutation has a stabilizing effect. This mutation confers a selective advantage during infection and suggests that P323L likely played a critical role in the rapid early emergence of the P323L/D614G genotype in the human population [133]. Additionally, the NSP12 P323L mutation, along with the P323L/G671S mutations, enhances the stability of the NSP12-NSP7-NSP8 complex, resulting in elevated viral RdRp activity [134]. 

## 5. Conclusions

Mutations in SARS-CoV-2 have significant implications for the COVID-19 pandemic. Our investigation revealed that NSP12-P323L, ORF9c-G50N, NSP14-I42V, membrane-A63T, Q19E, and NSP3-G489S are the most frequent mutations among Iranian SARS-CoV-2 sequences. These mutations can alter the sequence of primers and probes used in PCR-based tests, potentially leading to false-negative results. Additionally, mutations such as ORF9c G50N, ORF3a-T24I, NSP3-G489S, NSP5-P132H, NSP14-I42V, envelope-T9I, nucleocapsid-D3L, membrane-Q19E, and membrane-A63T might be responsible for the surge in the SARS-CoV-2 Omicron variant wave in Iran. Monitoring mutations in the SARS-CoV-2 genome can help anticipate future viral drug resistance. Furthermore, our study found that NSP12 mutation P323L, spike mutation D614G, and NSP14 mutation I42V stabilize the protein’s structure. Structure-based drug discovery holds promise as a therapeutic approach for treating virus infections by targeting specific molecular targets. Additionally, mutations in proteins like S and N may impact vaccine efficacy against novel mutations. Therefore, designing a novel multi-peptide subunit-based epitope vaccine candidate that targets conserved and hotspot regions in SARS-CoV-2 genomes is crucial for combating COVID-19.

## Figures and Tables

**Table 1:** Most common mutations in SARS-CoV-2 genome.

SARS-CoV-2 Gene	Mutation	Iran (%)	Worldwide (%)	*p*-Value
NSP12	P323L	88.2	99.43	0.0000 × 10
ORF9c	G50N	78.5	64.45	4.5540 × 10^−61^
NSP4	T492I	68.4	79.45	8.5418 × 10^−51^
NSP14	I42V	65.2	55.56	4.8895 × 10^−28^
M	A63T	64.5	46.32	4.8683 × 10^−98^
M	Q19E	63.7	46.05	1.8119 × 10^−92^
NSP3	G489S	61.7	40.29	2.3184 × 10^−88^
NSP3	T24I	61.6	40.23	4.0781 × 10^−88^
E	T9I	61.6	51.30	7.8620 × 10^−33^
NSP5	P132H	59.3	51.26	4.2737 × 10^−18^
N	R203K	52.1	23.72	1.6127 × 10^−162^
N	G204R	51.9	22.48	2.4319 × 10^−181^
S	D614G	50.9	97.59	0.0000 × 10
ORF3a	T223I	47.6	36.33	6.3225 × 10^−41^
NSP13	R392C	46.9	35.76	3.7519 × 10^−40^
NSP15	T112I	44.4	34.60	1.0122 × 10^−30^
NSP4	L264F	42.9	35.81	7.6654 × 10^−16^
NSP4	T327I	40.9	35.78	6.3115 × 10^−9^

**Table 2:** The stability and flexibility of SARS-CoV-2 mutation.

Mutation	ΔΔG	ΔΔG ENCoM	ΔΔG mCSM	ΔΔG SDM	ΔΔG DUET	ΔΔS_Vib_ ENCoM
**NSP12 P323L**	1.532	0.559	−0.264	0.700	0.118	−0.699
**ORF9c-G50N**	0.074	−0.010	−0.501	0.130	−0.294	0.012
**NSP14-I42V**	0.269	0.089	−0.809	−0.130	−0.678	−0.111
**S-D614G**	0.299	−0.048	−0.210	2.330	0.475	0.060

## Data Availability

The raw data supporting the conclusions of this article are available in Supplementary Files.

## Supplementary Materials

The following supporting information can be downloaded at: https://www.mdpi.com/article/10.3390/v16081331/s1, Table S1: Common mutations in the E protein among Iranian SARS-CoV-2 sequences. Table S2: Common mutations in the M protein among Iranian SARS-CoV-2 sequences. Table S3: Common mutations in the N protein among Iranian SARS-CoV-2 sequences. Table S4: Common mutations in the S protein among Iranian SARS-CoV-2 sequences. Table S5: Common mutations were identified among Iranian SARS-CoV-2 sequences in different COVID-19 waves. Table S6: The other amino acid substitutions in 18 SARS-CoV-2 mutations around the world. Table S7: The other amino acid substitutions in 18 SARS-CoV-2 mutations in Iran. File S1: Conserved regions and common mutations in other SARS-CoV-2 genes.
