# Supplementary material for: Comparative Atlas of SARS-CoV-2 Substitution Mutations: A Focus on Iranian Strains Amidst Global Trends
Source: Viruses. 2024 Aug 20;16(8):1331. doi: 10.3390/v16081331 (PMC11359407; doi:10.3390/v16081331)
Supplement: Supplementary file 1 [file viruses-16-01331-s001.zip › iran sars-cov-2 supp/Supplementary 8.docx]

**ORF1ab polyprotein**

The ORF1a polyprotein of SARS-CoV-2 encompasses 11 NSP proteins, NSP1, NSP2, NSP3, NSP4, 3CL-like protease, NSP6, NSP7, NSP8, NSP9, NSP10, and NSP11. SARS-CoV-2 NSP1, a virulence factor limiting protein synthesis by attaching directly to the human ribosome, suppresses the translation of host messenger RNAs (mRNAs). In our study, analyses suggest that the SARS-CoV-2 is highly conserved in the NSP1 and common mutations in NSP1 were S135R (34.1%) and R24C (0.26%) (Figure 4). S135R mutation was observed in all all VOCs [1] and may affect the interaction and function of the N- and C-terminal domains [2]. The R24C was the most common mutation in the NSP1 of SARS-CoV-2 in the US and worldwide (1.3%) which has been suggested that destabilizing effect on the protein [3]. Ghaleh et al analyzed 6,510,947 sequences of NSP1 in the six continents and revealed that E87D H110Y and R24C as a frequent mutations [4].

NSP2 is involved in coronavirus genome replication, SARS-CoV-2 infection and propose as a drug target for anti-SARS-CoV-2 drug development [5]. The structure and function of NSP2 in the SARS-CoV-2 have remained unknown [6]. Our study showed that the top mutations detected in NSP2 were V198I (9.10%), R27C (3.19%), and Y49C (2.95%). In the study of Cornillez-Ty et al. on the SARS-CoV, the deletion of NSP2 results in a modest reduction in viral titers [7]. Previous studies have been reported that SARS-CoV NSP2 interacts with prohibitin 1 (PHB1) and PHB2, which play a role in several cellular functions, including cell cycle, mitochondrial functions, and apoptosis [7, 8]. In addition, R27C was detected in 837 Indian SARS-CoV-2 strains[9]. Wang et al. reported that T85I may destabilize the structure of NSP2 but does not change the flexibility of NSP2 too much [10]. V198I was detected in India and was present in America, Europe, and Asia [9]. Gupta et al. studied a structure of SARS-CoV-2 NSP2 with cryo-EM experimental data and AlphaFold2 prediction. They suggested NSP2 interactions with endosomes, modulators of translation, and ribosomal RNA [11].

NSP3, also known as papain-like protease, the largest NSPs, plays a critical role in viral replication and function as a protease. In our study, just 11.3% of NSP3 was conserved, and the top mutations were NSP3-G489S (61.7%), NSP3-T24I (61.6%), A1006I (32.1%), and A488S (9.83%) (Figure 4). Vilar et al. analyzed 290,000 worldwide SARS-CoV-2 sequences from December 2019 to 30 December 2020. In this study, the mutation rate for T183, I1412, and A890 was 0.075 [12]. I1412T and T183I were detected in 420 SARS-CoV-2 variants from global areas in January–March 2021[13, 14]. Furthermore, S126L and T33I were observed in SARS-CoV-2 variants from global areas and in Serbia, respectively [13, 15]. Basheer et al. investigated a total of 1468 whole-genome sequences of the Omicron SARS-CoV-2 subvariant, BA.2.75, and found that the mutations G489S, T241I, S403L, and T183I had a frequency of 99% [16].

NSP3 with NSP4 rearrange host-derived membranes and plays a role in the replication of SARS-CoV [17]. A total of 23.8% of NSP4 was conserved (Figure 4), and several common mutations were observed, including T492I (68.4%), L264F (42.9%), and T327I (40.9%), which have been reported in previous studies [9, 18, 19]. T492I, L264F, L438F in NSP4 were reported in Omicron SARS-CoV-2 subvariant, BA.2.75 [16].

NSP5 encodes chymotrypsin-like main protease and is essential for viral assembly and maturation of SARS-CoV-2. P132H (59.3%), K90R (1.36%), and T255I (0.30%) are more frequently mutations that found in our samples. The main protease of Omicron SARS-CoV-2 has a mutation at position 132 (P132H) that reduces its thermal stability [20]. K90R and L205V mutations in NSP5 were associated with positive selection in Brazil [21]. K90R was a frequent mutation in 675 sequences in India from March 2020 to April 2021 and the top mutation in the world [22]. G15S and K90R are also found in European, Chinese, and Icelandic strains [23]. The study of Fung et al. revealed that G15S and K90R suppressed IFN-β production [24]. SARS-CoV-2 NSP5 can induce the expression of cytokines IL-1β, IL-6, TNF-α, and IL-2 in Calu-3 and THP1 cells. Furthermore, they showed that NSP5 could activate the NF-κB signaling pathway and enhances cytokine expression [25].

NSP6 is a membrane protein of approximately 34 kDa and can be found in many coronaviruses. NSP6, NSP3 and NSP4 form a protein complex in the endoplasmic reticulum to create double-membrane vesicles, which are required to develop the virus replication/transcription complex [26]. In our study, T77A (35.1%), L37F (9.38%), and Y80C (5.18%) were common mutations in NSP6. During the fourth wave of the pandemic in Pakistan, the T77A mutation was found to have the highest frequency [27]. The epidemiological analysis reported that the L37F mutation was associated with asymptomatic SARS-CoV-2 infection[28]. The transcriptome analysis of Sun et al. demonstrated that the L37F mutant failed to trigger pyroptosis and the downstream inflammasome pathway [29]. In our recent study, we showed that the T77A mutation is one of the top mutations globally, with a frequency of 69.8% [19].

The coronavirus RdRP machinery, which mediates viral replication, comprises of three proteins: NSP7, NSP8, and NSP12. The most common mutations in the NSP7 were M3I (0.26%) and M62I (0.20%). NSP8 presents different point mutations, including N118S (2.13%), T187I (0.50%) and A14T (0.38%). N118S had 97% incidence in the global BA.2.75 variant [16]. A14 to S and V were reported in 9692 SARS-CoV-2 [30].

In the RNA-synthesizing machinery, NSP9 is an essential RNA binding subunit. This protein form part of the replication and transcription complex (RTC) mediates all virus replication, overall pathogenicity, and viral genomic RNA reproduction. T35I (1.92%) mutation was the most common mutation in our samples.

NSP10 has 139 residues, two zinc fingers, by interaction with NSP14 and NSP16 activates numerous replication enzymes [31, 32]. The result of our study showed that the most common mutation in this region was T102I (0.20%). Rogstam et al. showed that the crystal structure of the unbound form of SARS-CoV-2 NSP10 is similar to SARS-CoV NSP10 [33]. Furthermore, a recent study suggests that NSP10 has a role in SARS-CoV-2 replication machinery and that the nsp10–nsp14 complex has a relatively weak affinity [34].

The NSP11 comprises 13–23 residues in different CoVs species. NSP11 was the most conserved region in Iranian SARS-CoV-2 (99.9 %) and S6L (0.26%) was the most common mutation in this region. Consistent with our results, NSP11 presents no mutations compared with the Wuhan-2019 virus ‘EPI_ISL_402124’ in Brazil [21].

NSP13 of SARS-CoV-2 is a superfamily 1 helicase that unwinds a double-stranded RNA or DNA, and with NSP1 block interferon activation [35]. NSP13 inhibits type I interferons (IFN-I)  production and enables SARS-CoV-2 to evade the host innate immune response [36]. Our analysis showed that the most commonly occurring NSP13 mutations were R392 (46.9%), T127N (15.6%) and P77L (7.66%). P77L was detected in the Delta variant and is one of the most frequently observed mutations in the world. This mutation increases the function of the NSP13 protein by binding to TBK1 and might suppress IFNß production, thereby making the SARS-CoV-2 more pathogenic [37]. Furthermore, P77L increases the function of the NSP13 protein [37].

NSP14 plays a critical role in viral RNA 5′ capping and has proofreading capability [38, 39]. Recent studies report that overexpression of NSP14 induces a near-complete shutdown in cellular protein synthesis [40]. The expression of Nsp14 leads to changes in the splicing patterns of multiple genes, enhances the production of circular RNAs (circRNAs), and triggers the activation of the NFkB pathway [41]. In our study, NSP14 was 24.9% conserved, and the top mutation in this region was I42V (65.2%) and A394V (7.48%). A394V was a positive selection site in the SARS-CoV-2 B.1.617 and is a common mutation globally [42].

NSP15 is conserved throughout the coronaviridae family, and its endonuclease function is essential for viral survival. According to recent findings, NSP15 nuclease activity is critical for evading host immune responses and could be a suitable target for an antiviral small molecule inhibitor [43, 44]. NSP15 was 50.7 % conserved and T112I (44.4 %), V127F (0.57 %), and H234Y (0.50 %) mutations were observed in our samples. In a study by Jung et al., it was found that T112I is one of the amino acid changes specific to the 21L clade of the Omicron variant [45]. H234Y was also observed in SARS‐CoV‐2 circulating during the third wave of the pandemic in Pakistan [46].

The transfer of a methyl group from S-adenosyl methionine (SAM) to RNA substrates is catalyzed by NSP16 [47]. Previous studies indicate that the NSP10-NSP16 complex is critical for CoVs’ viral replication [32, 48]. In our study, NSP16 was 90.8% conserved, and the top mutations were K160R (5.05 %) and L126F (0.87%). The first occurrence for the K160R mutation in P.1 (Gamma) variant was reported from Brazil [49].

**ORF3a protein**

The ORF3a protein composes of 275 amino acids that cause cytokine production and tissue inflammation. Our analysis revealed top mutations T223I (47.6%), S26L (8.79%), and Q57H (5.79%), on the ORF3a. The Q57H mutation was reported in Brazil and the United States. The amino acid glutamine (Q) changes with an amino acid histidine (H) in this mutation. Wang et al. suggested that the Q57H mutation makes the SARS-CoV-2 more infectious in the United States [50]. S26L is one of the frequent mutations globally and is detected in 15,928 worldwide SARS-CoV-2 sequences [51].

**ORF6 protein**

ORF6 is an ER/Golgi membrane protein that prevents the nuclear import complex from forming. ORF6 is a small protein with 61 residues and a molecular weight of about 7 kDa. In a dose-dependent way, Selinexor, a specific inhibitor of nuclear export, reduced SARS-CoV-2 ORF6-induced cellular toxicity [52]. In our sample D61L (23.5%) mutations were common mutations in this region. The characterized D61L mutation found in Omicron BA.2 and BA.4 shows a correlation with reduced IFN-β secretion by BA.2 in vitro, which is associated with the presence of the ORF6 [53]. This mutation is capable of interfering with the functions of the ORF6 protein at the NPC, leading to impaired innate immune evasion strategies of SARS-CoV-2 [54].

**ORF7a protein**

The accessory ORF7a is composed of 121 amino acids and facilitates infection and pathogenesis. The rennet study indicated that ORF7 plays a role as an immunomodulating factor for human CD14+ monocytes [55]. While another study reported that ORF7a of SARS-CoV inhibits cellular translation [56]. According to our results, common mutations T120I (4.56%), V82A (4.34%), and S83L (3.51%), were observed in Iran samples. S83L was also detected in North America and Oceania [57]. S83L also was observed SARS-CoV-2 mutations in clinical samples [58]. T39I is a frequently occurring mutation in the world [59]. Two common mutations V82A and T120I in ORF7a were also found in Pakistan and the USA, respectively [46, 60].

**ORF7b protein**

The function of SARS-CoV-2 ORF7b is not well investigated in SARS-CoV-2. ORF7b is located in the membrane of the endoplasmic reticulum ER of SARS-CoV-2, contains a transmembrane region, and functions in innate and adaptive immunity [61, 62]. Recent studies have shown that ORF7b may accelerate TNF-induced apoptosis in HEK293T cells and Vero E6 cells [63]. In our study, the most common mutation in ORF7b was T40I (7.73%) which was also observed in SARS-CoV-2 sequences from 45 countries [64].

**ORF8 protein**

ORF8 is a 121 amino-acid protein that functions in viral pathogenicity and replication in the host via the interferon pathway. Top mutations in ORF8 were R52I (0.77%), I121L (0.45%), A65D (0.27%). In addition to the United States, V62L has been identified in 11 other nations. In the United States, two high-frequency mutations, L84S and S24L, were discovered [50]. I121L was observed in asymptomatic Indian individuals [65].

**ORF9b protein**

ORF9b is located within the N gene, which codes for a 97 amino acid long protein localized in the mitochondrial membrane. T60A (15.8%) and P3L (6.60%) were common mutations in our study. T60A was observed in Delta and Delta Plus SARS-CoV-2 genomes and is common in worldwide SARS-CoV-2. ORF9b target Tom70, a mitochondrial import receptor that plays an important role in innate immune signaling [66]. Furthermore, ORF9, by mediating ATG5 induced autophagy in host cells [67]. The antibody responses were identified for ORF9b in COVID-19 convalescent patients [68].

**ORF9c protein**

ORF9c formerly also called ORF14 is a conserved region that presents in previous SARS-CoV [69]. In our study, ORF9c was 11.3% conserved and G50N (78.5%), G50W (8.14%), and V49L (1.04%) were the most common mutations in this region. ORF9c targeted Nod-like receptor NLRX1, proteinase-activated receptor 2 (F2RL1), and Nedd4 Family Interacting Protein 2 (NDFIP2) genes in the NF-κB pathway [66]. ORF9c interacts with membrane-related proteins, proteins in the mitochondria and endoplasmic reticulum (ER), and golgi that play a role in the protein biosynthesis and transport systems, affecting immune evasion, virulence, and pathogenesis [69]. G50N observed SARS-CoV-2 variants from the Philippines [70].

1. Shen, M., et al., *The antiviral activity of a small molecule drug targeting the NSP1-ribosome complex against Omicron, especially in elderly patients.* Frontiers in Cellular and Infection Microbiology, 2023. **13**: p. 193.

2. Hossain, A., et al., *Unique mutations in SARS-CoV-2 omicron subvariants' non-spike proteins: Potential impact on viral pathogenesis and host immune evasion.* Microbial pathogenesis, 2022: p. 105699.

3. Mou, K., et al., *Emerging Mutations in Nsp1 of SARS-CoV-2 and Their Effect on the Structural Stability.* Pathogens, 2021. **10**(10): p. 1285.

4. Ghaleh, S.S., et al., *SARS‐CoV‐2 Non-structural protein 1 (NSP1) mutation virulence and natural selection: Evolutionary trends in the six continents.* Virus Research, 2023. **323**: p. 199016.

5. Zheng, Y.-X., et al., *Nsp2 has the potential to be a drug target revealed by global identification of SARS-CoV-2 Nsp2-interacting proteins.* Acta Biochimica et Biophysica Sinica, 2021.

6. Mariano, G., et al., *Structural characterization of SARS-CoV-2: Where we are, and where we need to be.* Frontiers in molecular biosciences, 2020: p. 344.

7. Cornillez-Ty, C.T., et al., *Severe acute respiratory syndrome coronavirus nonstructural protein 2 interacts with a host protein complex involved in mitochondrial biogenesis and intracellular signaling.* Journal of virology, 2009. **83**(19): p. 10314-10318.

8. von Brunn, A., et al., *Analysis of intraviral protein-protein interactions of the SARS coronavirus ORFeome.* PloS one, 2007. **2**(5): p. e459.

9. Sarkar, R., et al., *Comprehensive analysis of genomic diversity of SARS-CoV-2 in different geographic regions of India: an endeavour to classify Indian SARS-CoV-2 strains on the basis of co-existing mutations.* Archives of Virology, 2021. **166**(3): p. 801-812.

10. Wang, R., et al., *Analysis of SARS-CoV-2 mutations in the United States suggests presence of four substrains and novel variants.* Communications biology, 2021. **4**(1): p. 1-14.

11. Gupta, M., et al., *CryoEM and AI reveal a structure of SARS-CoV-2 Nsp2, a multifunctional protein involved in key host processes.* bioRxiv, 2021.

12. Vilar, S. and D.G. Isom, *One year of SARS-CoV-2: How much has the virus changed?* Biology, 2021. **10**(2): p. 91.

13. Bui, N.-N., et al., *Haplotype distribution of SARS-CoV-2 variants in low and high vaccination rate countries during ongoing global COVID-19 pandemic in early 2021.* Infection, Genetics and Evolution, 2022. **97**: p. 105164.

14. Bitew, M., et al., *SARS-CoV-2 Genome Sequence Obtained from Ethiopia.* Microbiology Resource Announcements, 2022. **11**(2): p. e01182-21.

15. Miljanovic, D., et al., *The first molecular characterization of Serbian SARS-CoV-2 isolates from a unique early second wave in Europe.* Frontiers in microbiology, 2021. **12**: p. 1526.

16. Basheer, A., I. Zahoor, and T. Yaqub, *Genomic architecture and evolutionary relationship of BA. 2.75: A Centaurus subvariant of Omicron SARS-CoV-2.* Plos one, 2023. **18**(5): p. e0281159.

17. Sakai, Y., et al., *Two-amino acids change in the nsp4 of SARS coronavirus abolishes viral replication.* Virology, 2017. **510**: p. 165-174.

18. Tiwari, M. and D. Mishra, *Investigating the genomic landscape of novel coronavirus (2019-nCoV) to identify non-synonymous mutations for use in diagnosis and drug design.* Journal of Clinical Virology, 2020. **128**: p. 104441.

19. Fooladinezhad, H., et al., *SARS-CoV-2 NSP3, NSP4 and NSP6 mutations and Epistasis during the pandemic in the world: Evolutionary Trends and Natural Selections in Six Continents.* medRxiv, 2022.

20. Sacco, M.D., et al., *The P132H mutation in the main protease of Omicron SARS-CoV-2 decreases thermal stability without compromising catalysis or small-molecule drug inhibition.* Cell research, 2022. **32**(5): p. 498-500.

21. Timmers, L.F.S.M., et al., *SARS-CoV-2 mutations in Brazil: from genomics to putative clinical conditions.* Scientific reports, 2021. **11**(1): p. 1-14.

22. Yashvardhini, N., A. Kumar, and D.K. Jha, *Analysis of SARS-CoV-2 mutations in the main viral protease (NSP5) and its implications on the vaccine designing strategies.* Vacunas, 2021.

23. Yuan, F., et al., *Global SNP analysis of 11,183 SARS‐CoV‐2 strains reveals high genetic diversity.* Transboundary and emerging diseases, 2021. **68**(6): p. 3288-3304.

24. Fung, S.-Y., et al., *SARS-CoV-2 main protease suppresses type I interferon production by preventing nuclear translocation of phosphorylated IRF3.* International Journal of Biological Sciences, 2021. **17**(6): p. 1547.

25. Li, W., et al., *SARS-CoV-2 Nsp5 Activates NF-κB Pathway by Upregulating SUMOylation of MAVS.* Frontiers in immunology, 2021. **12**: p. 750969-750969.

26. Angelini, M.M., et al., *Severe acute respiratory syndrome coronavirus nonstructural proteins 3, 4, and 6 induce double-membrane vesicles.* MBio, 2013. **4**(4): p. e00524-13.

27. Anwar, M.Z., et al., *Coronavirus genomes and unique mutations in structural and non-structural proteins in Pakistani SARS-CoV-2 delta variants during the fourth wave of the pandemic.* Genes, 2022. **13**(3): p. 552.

28. Wang, R., et al., *Decoding asymptomatic COVID-19 infection and transmission.* The journal of physical chemistry letters, 2020. **11**(23): p. 10007-10015.

29. Sun, X., et al., *SARS-CoV-2 non-structural protein 6 triggers NLRP3-dependent pyroptosis by targeting ATP6AP1.* Cell Death & Differentiation, 2022: p. 1-15.

30. Reshamwala, S.M., et al., *Mutations in SARS‐CoV‐2 nsp7 and nsp8 proteins and their predicted impact on replication/transcription complex structure.* Journal of medical virology, 2021. **93**(7): p. 4616-4619.

31. Rosas-Lemus, M., et al., *The crystal structure of nsp10-nsp16 heterodimer from SARS-CoV-2 in complex with S-adenosylmethionine.* BioRxiv, 2020.

32. Chen, Y., et al., *Biochemical and structural insights into the mechanisms of SARS coronavirus RNA ribose 2′-O-methylation by nsp16/nsp10 protein complex.* PLoS pathogens, 2011. **7**(10): p. e1002294.

33. Rogstam, A., et al., *Crystal structure of non-structural protein 10 from severe acute respiratory syndrome coronavirus-2.* International journal of molecular sciences, 2020. **21**(19): p. 7375.

34. Kozielski, F., et al., *Identification of fragments binding to SARS-CoV-2 nsp10 reveals ligand-binding sites in conserved interfaces between nsp10 and nsp14/nsp16.* RSC Chemical Biology, 2022.

35. Vazquez, C., et al., *SARS-CoV-2 viral proteins NSP1 and NSP13 inhibit interferon activation through distinct mechanisms.* PLoS One, 2021. **16**(6): p. e0253089.

36. Sui, C., et al., *SARS-CoV-2 NSP13 Inhibits Type I IFN Production by Degradation of TBK1 via p62-Dependent Selective Autophagy.* The Journal of Immunology, 2022. **208**(3): p. 753-761.

37. Farooq Rashid, M.S., et al., *Structural Analysis on the Severe Acute Respiratory Syndrome Coronavirus 2 Non-structural Protein 13 Mutants Revealed Altered Bonding Network With TANK Binding Kinase 1 to Evade Host Immune System.* Frontiers in Microbiology, 2021. **12**.

38. Pachetti, M., et al., *Emerging SARS-CoV-2 mutation hot spots include a novel RNA-dependent-RNA polymerase variant.* Journal of translational medicine, 2020. **18**(1): p. 1-9.

39. Chen, Y., et al., *Functional screen reveals SARS coronavirus nonstructural protein nsp14 as a novel cap N7 methyltransferase.* Proceedings of the National Academy of Sciences, 2009. **106**(9): p. 3484-3489.

40. Hsu, J.C.-C., et al., *Translational shutdown and evasion of the innate immune response by SARS-CoV-2 NSP14 protein.* Proceedings of the National Academy of Sciences, 2021. **118**(24).

41. Zaffagni, M., et al., *SARS-CoV-2 Nsp14 mediates the effects of viral infection on the host cell transcriptome.* Elife, 2022. **11**: p. e71945.

42. Fan, L.-q., et al., *Biological Significance of the Genomic Variation and Structural Dynamics of SARS-CoV-2 B. 1.617.* Frontiers in Microbiology, 2021: p. 2837.

43. Hackbart, M., X. Deng, and S.C. Baker, *Coronavirus endoribonuclease targets viral polyuridine sequences to evade activating host sensors.* Proceedings of the National Academy of Sciences, 2020. **117**(14): p. 8094-8103.

44. Deng, X., et al., *Coronavirus endoribonuclease activity in porcine epidemic diarrhea virus suppresses type I and type III interferon responses.* Journal of virology, 2019. **93**(8): p. e02000-18.

45. Jung, C., et al., *Omicron: what makes the latest SARS-CoV-2 variant of concern so concerning?* Journal of virology, 2022. **96**(6): p. e02077-21.

46. Umair, M., et al., *Genomic surveillance reveals the detection of SARS‐CoV‐2 delta, beta, and gamma VOCs during the third wave in Pakistan.* Journal of Medical Virology, 2022. **94**(3): p. 1115-1129.

47. Snijder, E.J., et al., *Unique and conserved features of genome and proteome of SARS-coronavirus, an early split-off from the coronavirus group 2 lineage.* Journal of molecular biology, 2003. **331**(5): p. 991-1004.

48. Decroly, E., et al., *Crystal structure and functional analysis of the SARS-coronavirus RNA cap 2′-O-methyltransferase nsp10/nsp16 complex.* PLoS pathogens, 2011. **7**(5): p. e1002059.

49. Zimerman, R.A., et al., *Comparative genomics and characterization of SARS-CoV-2 P. 1 (gamma) variant of concern from Amazonas, Brazil.* Frontiers in medicine, 2022. **9**: p. 141.

50. Wang, R., et al., *Characterizing SARS-CoV-2 mutations in the United States.* Research square, 2020.

51. Azad, G.K. and P.K. Khan, *Variations in Orf3a protein of SARS-CoV-2 alter its structure and function.* Biochemistry and biophysics reports, 2021. **26**: p. 100933.

52. Lee, J.-G., et al., *Characterization of SARS-CoV-2 proteins reveals Orf6 pathogenicity, subcellular localization, host interactions and attenuation by Selinexor.* Cell & bioscience, 2021. **11**(1): p. 1-12.

53. Savellini, G.G., G. Anichini, and M.G. Cusi, *SARS-CoV-2 omicron sub-lineages differentially modulate interferon response in human lung epithelial cells.* Virus Research, 2023. **332**: p. 199134.

54. Kehrer, T., et al., *Impact of SARS-CoV-2 ORF6 and its variant polymorphisms on host responses and viral pathogenesis.* bioRxiv, 2022: p. 2022.10. 18.512708.

55. Zhou, Z., et al., *Structural insight reveals SARS-CoV-2 Orf7a as an immunomodulating factor for human CD14+ monocytes.* Iscience, 2021. **24**(3): p. 102187.

56. Addetia, A., et al., *Identification of multiple large deletions in ORF7a resulting in in-frame gene fusions in clinical SARS-CoV-2 isolates.* Journal of Clinical Virology, 2020. **129**: p. 104523.

57. Hassan, S.S., et al., *The importance of accessory protein variants in the pathogenicity of SARS-CoV-2.* Archives of Biochemistry and Biophysics, 2022: p. 109124.

58. Lau, B.T., et al., *Profiling SARS-CoV-2 mutation fingerprints that range from the viral pangenome to individual infection quasispecies.* Genome medicine, 2021. **13**(1): p. 1-23.

59. Yashvardhini, N., et al., *Genetic variations in the Orf7a protein of SARS-CoV-2 and its possible role in vaccine development.* Biomedical Research and Therapy, 2021. **8**(8): p. 4497-4504.

60. Rosenthal, S.H., et al., *Identification of eight SARS-CoV-2 ORF7a deletion variants in 2,726 clinical specimens.* bioRxiv, 2020.

61. Zhang, J., et al., *A systemic and molecular study of subcellular localization of SARS-CoV-2 proteins.* Signal transduction and targeted therapy, 2020. **5**(1): p. 1-3.

62. Stukalov, A., et al., *Multilevel proteomics reveals host perturbations by SARS-CoV-2 and SARS-CoV.* Nature, 2021. **594**(7862): p. 246-252.

63. Yang, R., et al., *SARS-CoV-2 accessory protein ORF7b mediates tumor necrosis factor-α-induced apoptosis in cells.* Frontiers in Microbiology, 2021. **12**.

64. Nguyen, T.T., et al., *Genomic mutations and changes in protein secondary structure and solvent accessibility of SARS-CoV-2 (COVID-19 virus).* Scientific Reports, 2021. **11**(1): p. 1-16.

65. Rangaiah, A., et al., *Whole-Genome Sequencing of SARS-CoV-2 Strains from Asymptomatic Individuals in India.* Microbiology Resource Announcements, 2022. **11**(2): p. e00850-21.

66. Gordon, D.E., et al., *A SARS-CoV-2 protein interaction map reveals targets for drug repurposing.* Nature, 2020. **583**(7816): p. 459-468.

67. Shi, C.-S., et al., *SARS-coronavirus open reading frame-9b suppresses innate immunity by targeting mitochondria and the MAVS/TRAF3/TRAF6 signalosome.* The Journal of Immunology, 2014. **193**(6): p. 3080-3089.

68. Jiang, H.-w., et al., *Global profiling of SARS-CoV-2 specific IgG/IgM responses of convalescents using a proteome microarray.* MedRxiv, 2020.

69. Andres, A.D., et al., *SARS-CoV-2 ORF9c is a membrane-associated protein that suppresses antiviral responses in cells.* bioRxiv, 2020.

70. Velasco, J.M., et al., *Coding-complete genome sequences of 11 SARS-CoV-2 B. 1.1. 7 and B. 1.351 variants from metro Manila, Philippines.* Microbiology Resource Announcements, 2021. **10**(28): p. e00498-21.
